# Adiponectin reduces lipid content in chicken myoblasts by activating AMPK signaling pathway

**DOI:** 10.1042/BSR20212549

**Published:** 2022-06-06

**Authors:** Qingmei Hu, Dan Wang, Hai Lin, Haifang Li, Jingpeng Zhao, Hongchao Jiao, Xiaojuan Wang

**Affiliations:** 1 Faculty of Animal Science and Technology, Shandong Agricultural University, Shandong Provincial Key Laboratory of Animal Biotechnology and Disease Control and Prevention, Shandong Agricultural University, Taian, Shandong, P. R. China; 2 Faculty of Life Sciences, Shandong Agricultural University, Shandong Provincial Key Laboratory of Animal Biotechnology and Disease Control and Prevention, Shandong Agricultural University, Taian, Shandong, P. R. China

**Keywords:** adiponectin, AMPK, chicken myoblast, lipid metabolism

## Abstract

Studies in mammals have shown that adiponectin is secreted mainly by adipocytes, and it plays a crucial role in glucose and lipid metabolism in muscles. Clarifying the cross-talk role of adiponectin between adipose tissue and skeletal muscle tissue is very important for internal homeostasis. The glucose and lipid metabolism of chicken is different from that of mammals, and the role of adiponectin in chickens is unclear. Therefore, it is of great significance to study the effect and mechanism of adiponectin on lipid metabolism in chickens. In the present study, the regulating effect of adiponectin on lipid metabolism in chicken myoblasts was explored by adding a certain concentration of exogenous recombinant adiponectin. Results showed that adiponectin reduced intracellular lipid content, increasing the mRNA expression of adiponectin receptor and cellular uptake of glucose and fatty acids. In addition, adiponectin activated the 5′ adenosine monophosphate activated protein kinase (AMPK) signaling pathway. The above results suggested that adiponectin reduced intracellular lipid content, mainly by binding to adiponectin receptor, activating AMPK pathway, increasing cellular uptake of glucose and fatty acids and promoting lipid oxidation.

## Introduction

Adipocytes secrete abundant adipocytokines, one of which is adiponectin. Adiponectin has been shown to be widely expressed in bone marrow, osteoblasts, cardiomyocytes and salivary epithelial cells of mammals, as well as in liver, skeletal muscle, pituitary, hypothalamus and kidney tissues of poultry such as chickens and geese [1–3]. Adiponectin is a monomer glycoprotein with a molecular weight of about 30 ku and contains 22 (Gly-X-Pro and GLy-X-Y) repeats [4,5]. Adiponectin of human, mouse and poultry consists of 244, 247 and 245 amino acids, respectively [2,3]. The homology of adiponectin gene in human and mouse is higher than 80%, while the homology of adiponectin gene in poultry is only about 66% due to the great difference of adiponectin gene structure between birds and mammals [6–9]. Many studies in mammals and humans have demonstrated that adiponectin is involved in regulation of lipid and glucose metabolism [10]. In recent years, it has been reported that adiponectin in chicken is negatively correlated with abdominal fat deposition [11] and can inhibit fat deposition and promote mitochondrial biogenesis in adipocytes [12,13], thus further confirming its role in regulating lipid and glucose metabolism. Skeletal muscles use fatty acids and glucose as primary fuels. Compared with mammals, birds have higher levels of glucose and lower concentrations of insulin [14,15]. Broiler chicken of modern strain is characterized by a fast muscle growth rate, heavy body weight and high fat deposition [16]. These characteristics suggest that chickens have a more intractable insulin cascade and lipid metabolism in skeletal muscle tissues, and the study of the chicken model would be beneficial to our understanding of adiponectin.

Adiponectin receptor 1 (ADPNR1) and adiponectin receptor 2 (ADPNR2) are two distinct isoforms of adiponectin receptors bound by secretory adiponectin. These two isoforms can be expressed in different cell types, such as 3T3-L1 adipocytes, skeletal muscle and monocytic cells [17–19]. The expression of ADPNR1 was highest in skeletal muscle of human, mouse, pig and chicken, while the expression of ADPNR2 was highest in liver [20–22]. Studies have shown that ADPNR2 can regulate lipid and glucose metabolism, oxidative stress and inflammation [12,23,24].

Adiponectin is widely believed to be a key regulator of energy homeostasis through activation of peroxisome proliferator activated receptor (PPAR) and 5′ adenosine monophosphate activated protein kinase (AMPK) in skeletal muscle and liver of mammals [25]. AMPK is considered as a potential target of metabolic disorders because it is an important metabolic sensor that regulates cellular energy homeostasis [26]. It has been shown that activation of AMPK can decrease triglyceride and cholesterol synthesis and increase fatty acid oxidation in humans and animals [27,28]. Similarly, related functions have been reported in poultry fat cells [13]. Adenovirus-mediated adiponectin delivery has been reported to enhance AMPK activation in skeletal muscle [29,30]. It has also been reported that adiponectin regulates peroxisome proliferator-activated receptor coactivator-1α and mitochondria biogenesis by regulating calcium signaling and the ADPNR1-AMPK-silent mating type information regulation 2 homolog (SirT1) dependent pathway [31,32]. The functions of the AMPK pathway in chickens and mammals are similar [33]. We thus hypothesis that adiponectin is involved in the lipid metabolism in chicken muscles, through regulating AMPK pathways.

The purpose of the present study was to investigate the effect and mechanism of adiponectin on lipid metabolism of chicken muscles. The recombinant adiponectin protein was employed to chicken primary myoblasts to induce the adiponectin treatment. The lipid uptake, lipid accumulation, expressions of genes in lipid metabolism, AMPK signaling pathways were detected, and the involvement of AMPK was further determined using an inhibitor specific for AMPK.

## Materials and methods

### Preparation and identification of recombinant adiponectin protein

Recombinant adiponectin protein was prepared and identified by Jinan Boshang Biotechnology Co., Ltd., using an *Escherichia coli* expression system, according to the chicken adiponectin gene sequence (NM_206991).

### Animals

The fertile eggs were provided by a commercial source (Jinan SAIS Poultry Co., LTD., China) and incubated at 37°C under a relative humidity of 60–70%. The experimental treatment of chicken embryos was carried out in Shandong Agricultural University. All live embryos were euthanized by diethyl ether inhalation. All study procedures were approved by the Shandong Agriculture University Animal Care and Use Committee (SDAUA-2013-019) and were conducted in accordance with the Guidelines for Experimental Animals established by the Ministry of Science and Technology (Beijing, China).

### Cell culture and treatments

Cell culture: Primary cultures of chicken embryo myoblasts were prepared using a modified method described previously by Yablonka-Reuveni and Nameroff [34] and Wang [35]. In brief, myoblast cells were derived from the breast muscle tissues of 15-day-old embryos. The isolated myoblasts were cultured in Dulbecco’s modified Eagle's medium (DMEM; HyClone, Thermo Fisher, Shanghai, China) supplemented with 16% foetal bovine serum and 1% penicillin/streptomycin (Solarbio, Beijing, China) in a humidified 5% CO_2_ atmosphere at 37°C until the cells reached approximately 95% confluence.

Palmitic acid preparation: Palmitic acid was dissolved in 50% ethanol and 10 mol/L NaOH heated to 60°C to obtain a clear solution, which was then immediately mixed with fatty acid-free BSA.

Trial 1: Adiponectin treatment. Under 300 μM palmitic acid in serum-free medium for 12 h (PA) or no PA pretreatment, myoblasts were exposure to 5 μg/ml recombinant adiponectin for 12 h (ADPN) or no ADPN (Control). The cells were then rinsed with D-Hanks’ solution, collected and subjected to the further analysis.

Trial 2: Adiponectin and AMPK inhibitor treatment. All myoblasts were incubated for 12 h of 300 μM palmitic acid in serum-free medium (PA) prior to the treatment. Under 15 μM Compound C for 1 hour (CC) or no CC pretreatment, myoblasts were exposure to 5 μg/ml recombinant adiponectin for 12 h (ADPN) or no ADPN (Control). The cells were then rinsed with D-Hanks’ solution, collected and subjected to the further analysis.

### Cell viability assay

The Cell Counting kit-8 (CCK-8) assay was performed to investigate the viability of myoblasts cultured in 96-well plates. Briefly, 100 μl of CCK-8 solution at a 1:10 dilution was added to each well of the plate, and the plate was incubated at 37°C for 2.5 h. Absorbance was measured at 450 nm with a microplate reader (Elx808, Bio-Tek, Winooski, VT). The mean optical density (OD) of six wells for each indicated group was used to calculate the cell viability percentage.

### Oil Red O staining

According to the method of Lillie and Fullmer [36], the Oil Red O staining method was appropriately modified as described by Zhang et al. [37] and Zhao et al. [38]. Cells were washed twice with 1× PBS and subsequently fixed with 4% formaldehyde for 30 min. Following fixation, the cells were washed twice with 1× PBS and subsequently stained with 0.6% Oil Red O solution for 30 min. Haematoxylin staining was performed to visualize the cell nuclei. After washing, the cultures were photographed with an inverted microscope (Olympus, Japan).

### Detection of intracellular content

Triglyceride (TG) and total cholesterol (TCH) in chicken myoblasts were detected using commercial diagnostic kits (Nanjing Jiancheng Bioengineering Institute, China).

### Real-time PCR analysis

Total RNA extraction and qRT-PCR were performed as described previously [39–42]. The total RNA of myoblasts was extracted by TransZol Up (TransGen Biotech, China), the quantity of the RNA was measured by spectrophotometry (Eppendorf, Germany), and the quality of the RNA was verified by calculating the ratio between the absorbance values at 260 and 280 nm (A260/280 ≈ 1.8–2.1) as well as by electrophoresis. Next, reverse transcription was performed using total RNA (1 µg) for first-strand cDNA synthesis with the Transcriptor First Strand cDNA Synthesis Kit (Roche, Germany). The cDNA was amplified in a 20 µl PCR reaction system containing 0.2 µmol/L of each specific primer (Sangon, China) and the SYBR Green master mix (Roche, Germany) according to the manufacturer’s instructions. Real-time PCR was performed at the ABI QuantStudio 5 PCR machine (Applied Biosystems; Thermo, U.S.A.). Each cycle consisted of a 5 s denaturation step at 95°C, followed by a 34 s annealing and extension steps at 60°C. The primers sequences are shown in Table 1. The PCR products were verified by electrophoresis and DNA sequencing. Standard curves were generated using cDNA collected from the samples under assay, and the PCR data were analyzed using the 2^−ΔΔCT^ method [43–45]. The mRNA levels of the target genes were normalized to those of glyceraldehyde 3-phosphate dehydrogenase (GAPDH) and β*-*actin (ΔCT) [41,44,46,47]. All of the samples were run in duplicate, and the primers were designed to span an intron to avoid genomic DNA contamination.

**Table 1:** Gene-specific primers used for the analysis of chicken gene expression

Gene	Sequences (5′→3′)	Accession No.	Product size (bp)
*ADPN*	F: ACCCAGACACAGATGACCGTT	NM_206991	238
	R: GAGCAAGAGCAGAGGTAGGAGT		
*ADPNR1*	F: GGAGAAGGTTGTGTTTGGGATGT	NM_001031027	218
	R: TGGAGAGGTAGATGAGTCTTGGC		
*ADPNR2*	F: ACACACAGAGACTGGCAACATC	NM_001007854	144
	R: CCCAAGAAGAACAATCCAACAACC		
*ACC*	F: AATGGCAGCTTTGGAGGTGT	NM_205505	136
	R: TCTGTTTGGGTGGGAGGTG		
*FAS*	F: CTATCGACACAGCCTGCTCCT	J03860	107
	R: CAGAATGTTGACCCCTCCTACC		
*PPARα*	F: AGACACCCTTTCACCAGCATCC	AF163809	167
	R: AACCCTTACAACCTTCACAAGCA		
*PPARγ*	F: CCAGCGACATCGACCAGTT	AF163811	145
	R: GGTGATTTGTCTGTCGTCTTTCC		
*FATP1*	F: TCAGGAGATGTGTTGGTGATGGAT	DQ352834	138
	R: CGTCTGGTTGAGGATGTGACTC		
*ATGL*	F: AAGTCCTGCTGGTCCTCTCCTTG	NM_001113291.1	94
	R: AGTGTTGTCCTCCATCTGGTCCTC		
*GAPDH*	F: ACATGGCATCCAAGGAGTGAG	NM_204305.1	244
	R: GGGGAGACAGAAGGGAACAGA		
*β-Actin*	F: CTGGCACCTAGCACAATGAA	NM_205518.1	123
	R: CTGCTTGCTGATCCACATCT		

### Glucose and fatty acid uptake

Glucose uptake was measured by the uptake of fluorescently labeled deoxyglucose (2-NBDG [2-[N-(7-nitrobenz-2-oxa-1,3-diazol-4-yl) amino]-2-deoxy-D-glucose]) using Glucose Uptake Assay Kit (600470, Cayman Chemical Company, U.S.A.), according to the manufacturer’s instructions. After the experimental treatment, all culture medium was removed from each well and replaced with culture medium in the absence (negative control) or presence of 2-NBDG (final concentration of 200 µg/mL) and incubated at 37°C with 5% CO_2_ for 40 min before flow cytometry analysis.

Fatty acid uptake was assessed by the uptake of fluorescently labeled fatty acids using the QBT Fatty Acid Uptake Assay Kit (R8132, Molecular Devices, U.S.A.), according to the manufacturer’s instructions. After the experimental treatment, all culture medium was removed from each well and replaced with culture medium in the absence (negative control) or presence of fluorescent fatty acids (final concentration of 300 µg/ml) and incubated at 37°C with 5% CO_2_ for 10 min before flow cytometry analysis.

The uptake reaction was stopped by removing the incubation medium and washing the cells twice with pre-cold 1× PBS. Cells were collected by centrifugation at 3000 rpm for 5 min and subsequently resuspended in 400 µl pre-cold 1×PBS and maintained at 4°C for later flow cytometry analysis performed within 30 min. For each measurement, data from 10,000 single cell events was collected using a BD LSRFortessa flow cytometer (BD Biosciences, San Jose, CA, U.S.A.), and each flow cytometric measurement was digitized and quantified as fluorescence intensity following corrected by its negative control.

### Western blot

Cells were homogenized in lysis buffer (Beyotime, Jiangsu, China) and centrifuged at 12,000×***g*** for 10 min at 4°C. The supernatant was collected, and proteins were quantified using a BCA protein assay kit (Beyotime, Jiangsu, China) according to the manufacturer’s protocol. Eighteen-microgram aliquots of protein were electrophoresed on a 7.5% SDS polyacrylamide gel, and separated proteins were transferred to a polyvinylidene fluoride membrane in western transfer buffer. The membrane was blocked prior to incubation with the following primary antibodies which were purchased from CST (Boston, MA, U.S.A.) with a dilution of 1:1000: anti-phospho-AMPKα (Thr 172) (Rabbit, #2531), anti-AMPKα (Rabbit, #2532), anti-phospho-acetyl CoA carboxylase (ACC) (Ser79) (Rabbit, #3661), anti-ACC (Rabbit, #3662) and anti-carnitine palmitoyl transferase 1 (CPT1) (Rabbit, #12252). Protein detection was performed using horseradish peroxidase-linked anti-rabbit secondary antibodies (A0208, 1:1000, Beyotime, Shanghai, China) with enhanced chemiluminescence plus western blot detection reagents. These antibodies have previously been validated for use with chicken samples [18,37]. The films were scanned, and the intensities of specific bands were quantified using BioSpectrum 810 with VisionWorks LS 7.1 software (UVP LLC) [42,45]. Bands were normalized to β-actin (Beyotime, Shanghai, China) levels in the same sample. Protein molecular weight markers were used to verify the detected proteins.

### CPT1 activity

The enzyme activity of CPT1 was detected using ELISA kit (Shanghai Enzyme-linked Biotechnology Co. LTD, China).

### Statistical analysis

Data are presented as mean ± SEM. Statistical analysis of the data was performed by one-way ANOVA and SAS statistical software (SAS version 8e, SAS Institute, U.S.A.). *P*<0.05 were considered statistically significant and were considered to be approaching significance at *P*<0.10.

## Results

### Effects of adiponectin on the intracellular lipid content

**Figure 1:**
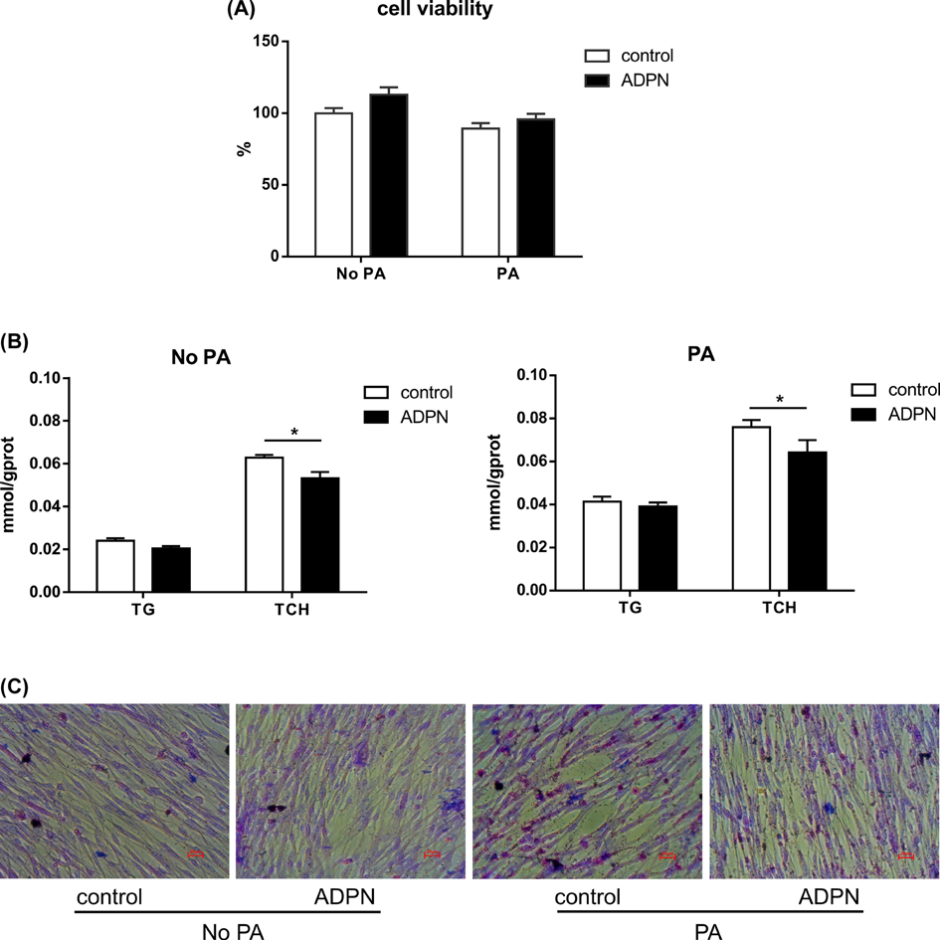
Effects of adiponectin on cell viability and intracellular lipid content in chicken myoblasts (**A**) Cell viability of myoblasts. (**B**) TG (triglyceride) and TCH (total cholesterol) in chicken myoblasts were detected by triglyceride reagent kit and total cholesterol reagent kit respectively. (**C**) Oil Red O staining of lipid droplets photographed at 400× magnification. Data are presented as mean ± SEM. *P<0.05; *n* = 6 in each group.

We first tested the effects of ADPN and PA on myoblasts viability, before investigating their effects on lipid metabolism in chicken myoblasts. We found that ADPN and PA did not significantly affect the viability of chicken myoblasts (*P*>0.05, Figure 1A). Next, we measured triglycerides and total cholesterol in myoblasts. It was found that ADPN significantly reduced the intracellular TCH content with and without PA pretreatment (Figure 1B, *P*<0.05). This result was further confirmed by the Oil Red O staining (Figure 1C).

### Effects of adiponectin on the uptake of glucose and fatty acids by chicken myoblasts

**Figure 2:**
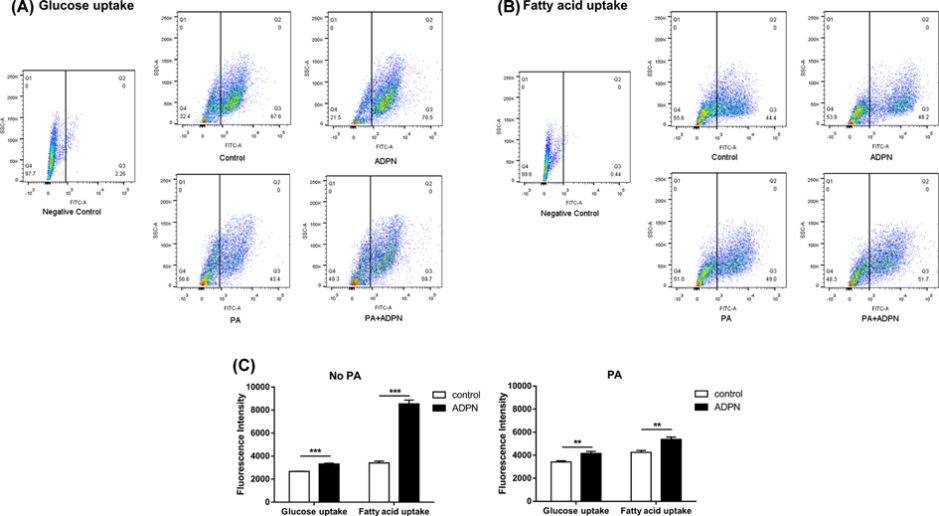
Effects of adiponectin on the glucose and fatty acid uptake by chicken myoblasts (**A–C**) The glucose and fatty acid uptake in chicken myoblasts were determined by flow cytometry. Data are presented as mean ± SEM. **P<0.01, and ***P<0.001. *n*=6 in each group.

Flow cytometry detection showed that ADPN treatment significantly (*P*<0.01) increased uptake of glucose and fatty acids by myoblasts, with and without PA pretreatment (Figure 2A–C).

### Effects of adiponectin on the genes expressions related lipid metabolism in chicken myoblasts

**Figure 3:**
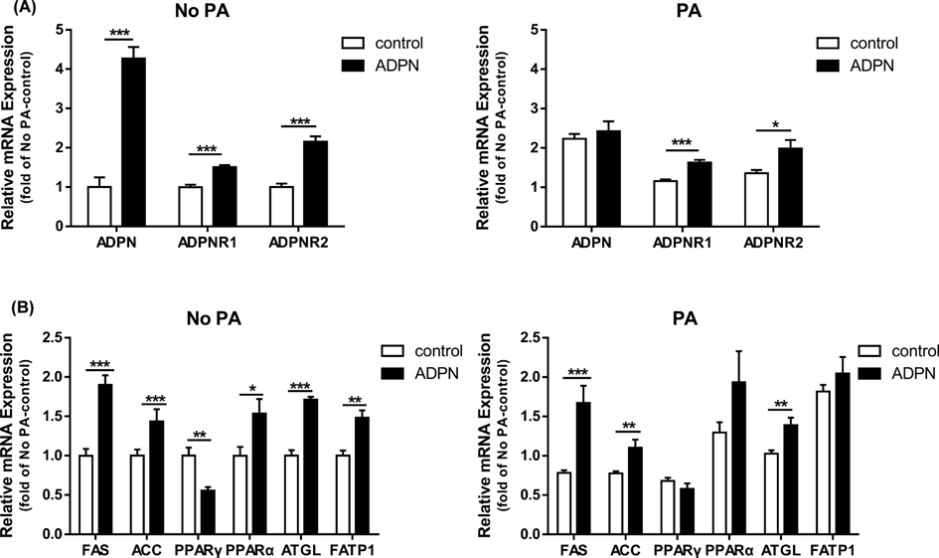
Effects of adiponectin on the mRNA expression of genes related to lipid metabolism in chicken myoblasts (**A** and **B**) The relative mRNA abundances of lipid metabolism genes were determined by real-time PCR. Data are presented as mean ± SEM; *P<0.05, **P<0.01 and ***P<0.001, *n*=6 in each group.

As shown in Figure 3A,B, the mRNA expression of *ADPN* was significantly (*P*<0.001) up-regulated by the addition of recombinant adiponectin, but it was not changed when myoblasts were pretreated with PA. However, the mRNA expressions of *ADPNR1* and *ADPNR2* were significantly (*P*<0.05) increased by either the addition of recombinant adiponectin alone or under palmitic acid pretreatment. Besides, we found that ADPN treatment significantly (*P*<0.05) up-regulated expressions of genes related fatty acids uptake (*FATP1*), genes related fat synthesis (*FAS* and *ACC*), genes related lipolysis and oxidation (*PPARα* and *ATGL*). The genes mentioned above were also significantly (*P*<0.01) up-regulated by ADPN under PA pretreatment, except for *PPARα* and *FATP1*. *PPARγ* was significantly (*P*<0.01) down-regulated by APDN without PA pretreatment.

### Involvement of the AMPK signaling pathway in the effects of adiponectin

**Figure 4:**
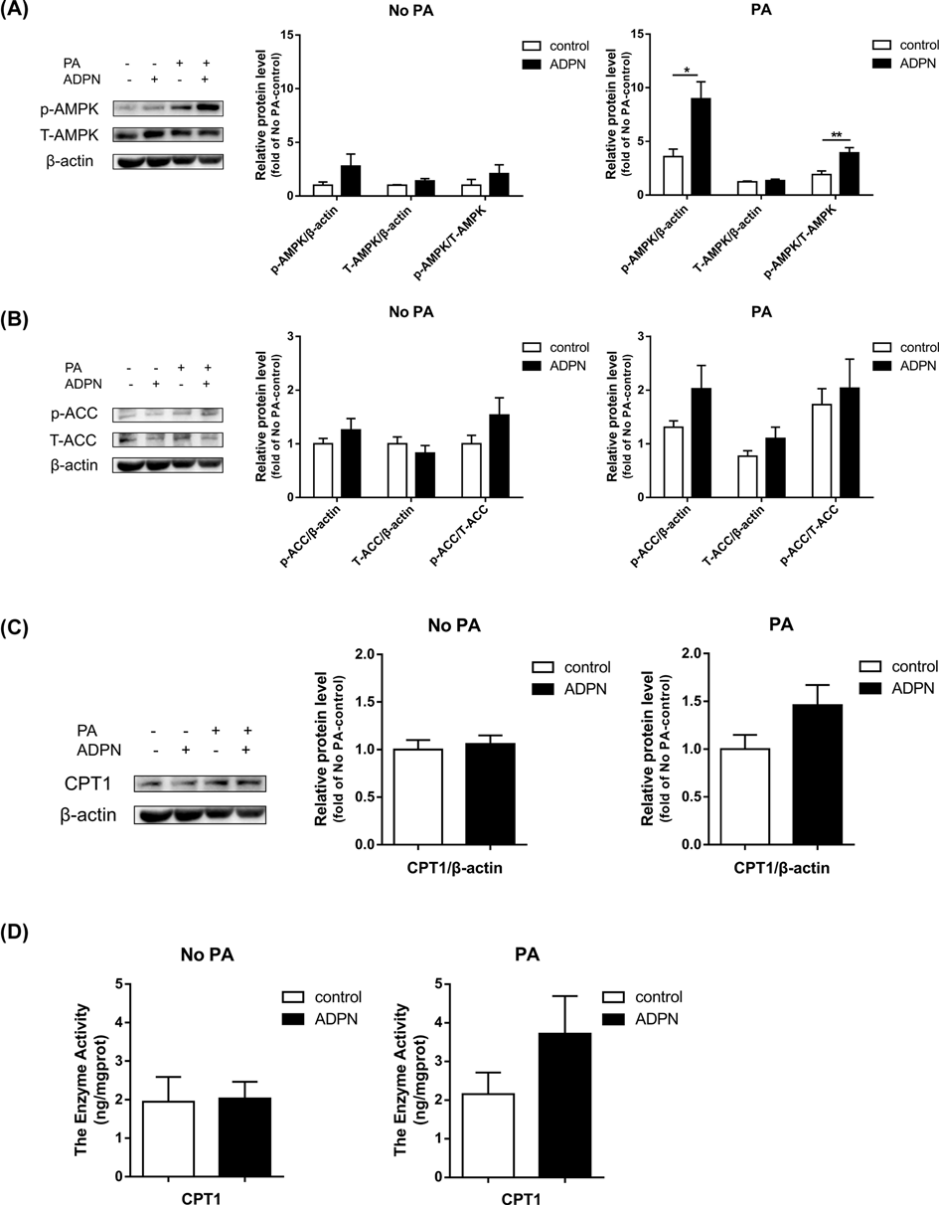
Adiponectin activated the AMPK/ACC/CPT1 signaling pathway in cultured chicken myoblasts (**A–C**) Representative bands of AMPK, ACC and CPT1. The protein expressions of AMPK, ACC, and CPT1 in cultured chicken myoblasts were determined by Western blot. (**D**) The enzyme activity of CPT1 was detected by ELISA kit. Data are presented as mean ± SEM; *P<0.05 and **P<0.01; *n*=6 in each group.

AMPK activation via increased Thr^172^ phosphorylation leads to inactivation of acetyl-CoA carboxylase (ACC1/ACC2) and the resulting fall in malonyl-CoA relieves inhibition of CPT1, which increased import and β-oxidation of free fatty acids into mitochondria [48]. Therefore, we measured protein expression of AMPK, ACC and CPT1 in myoblasts, to investigate the effects of ADPN on the fatty acids β-oxidation and the involvement of AMPK pathway. It was found that, when myoblasts were pretreated with PA, the AMPK phosphorylation was significantly (*P*<0.01) activated by ADPN. However, ACC phosphorylation, CPT1 protein and activity were not significantly (*P*>0.05) changed by ADPN (Figure 4A–D).

**Figure 5:**
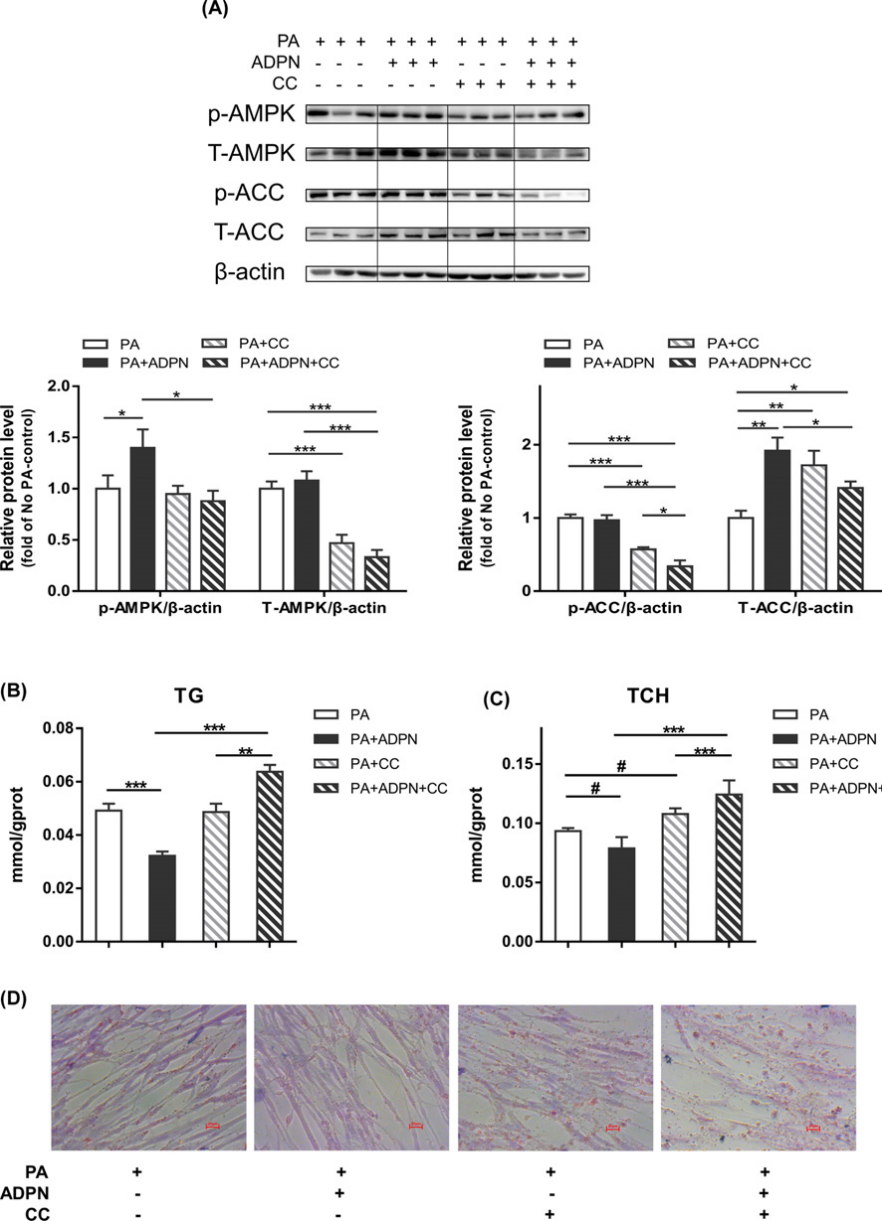
AMPK inhibitor Compound C reversed the effects of ADPN on lipid content in cultured chicken myoblasts (**A**) Representative bands of AMPK, ACC. The protein expressions of AMPK and ACC in cultured chicken myoblasts were determined by Western blot. (**B**) TG (triglyceride) in chicken myoblasts was detected by triglyceride reagent kit. (**C**) TCH (total cholesterol) in chicken myoblasts was detected by total cholesterol reagent kit. (**D**) Oil Red O staining of lipid droplets photographed at 400× magnification. Data are presented as mean ± SEM. #*P*<0.1, *P<0.05, **P<0.01 and ***P<0.001; *n*=6 in each group.

AMPK inhibitor, Compound C, was used to further verify the involvement of AMPK pathway in the ADPN effects. As Figure 5A shown, Compound C significantly inhibited the protein expression of AMPK (*P*<0.001). Adiponectin under PA pretreatment significantly activated the phosphorylation of AMPK, and the effect was impaired in the presence of Compound C (*P*<0.05). Adiponectin didn’t affect ACC phosphorylation (*P*>0.05) but increased the protein level of total ACC (*P*<0.01) which was alleviated in the presence of Compound C (*P*<0.05). The results showed that the addition of adiponectin under PA pretreatment significantly reduced TG (*P*<0.001) content and tended to reduce TCH (*P*<0.1) content. However, when AMPK was inhibited, the effects of ADPN on lipid content were reversed (Figure 5B,C). This result was further confirmed by the Oil Red O staining (Figure 5D).

## Discussion

### Adiponectin reduced intracellular lipid content without affecting chicken myoblasts viability

There was a negative correlation between serum adiponectin levels and liver lipid content in Japanese patients with Type 2 diabetes [49]. And research shows that adiponectin stimulates mitochondrial biogenesis and reduces lipid content in human and animal adipocytes [50]. In the present study, we found that recombinant adiponectin and palmitic acid treatment did not significantly affect the cell viability. In line with the previous study in human and mammals, our present study in cultured chicken myoblasts showed that the addition of 5 μg/ml ADPN reduced fat content in both basal and PA conditions, which is manifested as the reduction of TG and TCH content, as well as the decrease of lipid droplets. These results indicated that adiponectin at a certain concentration plays a lipid-lowering role in chickens.

### Both ADPNR1 and ADPNR2 are required for adiponectin function in chicken myoblasts

Secreted adiponectin binds to two distinct isoforms of receptors such as ADPNR1 and ADPNR2 [19]. Yamauchi et al. [19] confirmed that the binding of adiponectin to cell membrane is dependent on ADPNR1 and ADPNR2 in cultured cells, and the adiponectin binding and function is disrupted in ADPNR1/ADPNR2-double-knockout mice, demonstrating that adiponectin receptors represent key receptors *in vivo* [51,52]. We thus measured both ADPNR1 and ADPNR2 expressions in chicken myoblast. The results showed *ADPN* expression was significantly up-regulated by the addition of recombinant adiponectin only in non-PA conditions, while the ADPNR1 and ADPNR2 mRNA expressions were significantly increased with or without PA pretreatment. Although Kadowaki and Yamauchi [23] found that ADPNR1 was mainly expressed in muscle tissues in mice, the present experiment found that both ADPNR1 and ADPNR2 responded positively to adiponectin, which showed that both receptors play important roles in chicken myoblasts. Different from the previous study which showed that obesity decreased expression levels of ADPNR1/R2, thereby reducing adiponectin sensitivity [23], we did not find the reduction of ADPNR1/R2 by PA, this result may be due to the PA addition dose.

### Adiponectin promotes fat uptake, lipolysis and fat oxidation, thereby reducing fat deposition in chicken myoblasts

Adiponectin is considered as an important factor which plays a critical role in glucose as well as lipid metabolism [53]. In skeletal muscle, full-length or globular domain of adiponectin causes increase in glucose uptake by promoting the translocation of the glucose transporter 4 to the plasma membrane [48,54]. Accordingly, our present results showed that adiponectin promoted the uptake of glucose and fatty acids by chicken myoblasts. The expression of FATP1, a protein that facilitates the uptake of long-chain fatty acids (LCFAs) [55], was also increased by ADPN treatment in the present study. FATP1 is expressed in brown adipose tissue, white adipose tissue, skeletal muscle and heart, but in brain, lung, pancreas and kidney to a low degree [56–58], Stahl et al. [59] found that overexpression of FATP alone leads to an increase in LCFA uptake.

We further examined the genes involved in lipid metabolism. Fatty acid synthase (FAS), an essential multi-enzyme of 273 kDa polypeptide dimer complex, catalyzes the endogenous *de novo* synthesis of saturated fatty acids from simple molecular precursors such as acyl-CoA and malonyl-CoA [60–62]. Research showed that FAS could produce triglycerides, complex saturated lipids and lipoproteins (such as very low density lipoprotein and low density lipoprotein) [62,63]. However, skeletal muscle is an important site for glucose storage and lipid utilization rather than *de novo* synthesis of saturated fatty acids [64]. Although we found that ADPN treatment significantly up-regulated genes expression (*FAS* and *ACC*), it does not mean that their protein and enzyme activity level also increase.

We have known that PPARs which are transcription factors belonging to the nuclear receptor superfamily control expression of various genes that are crucial for lipid and glucose metabolism [65], and adiponectin were stimulated by PPARγ activation, improving insulin resistance and glucose homeostasis [66,67]. Therefore, the decreased PPARγ expression in the present study may be related to the negative regulation caused by ADPN overexpression. As PPARγ expression is greater in adipose tissue than in skeletal muscle [68], it is unclear whether PPARγ exerts direct effects on skeletal muscles or alters expression of adipocyte genes that convey signals to skeletal muscles. However, glucose homeostasis and lipid metabolism, which mainly occurs in the muscles, are the major mechanisms of PPARγ involved in the improvement of insulin resistance [69,70]. PPARα is expressed in numerous tissues in rodents and humans including liver, kidney, heart, skeletal muscle and brown fat [71,72]. The critical role of PPARα agonists in the regulation of β-oxidation of fatty acids has been well documented [65,73], and they also stimulate the cellular uptake of fatty acids by increasing the expression of the FATP and fatty acid translocase (FAT) [74]. In the heart, PPARα primarily supplies energy to the myocardium by regulating the genes responsible for fatty acid uptake and oxidation [75]. Therefore, the upregulated expressions of *PPARα* and *FATP1* as well as the increased fatty acids uptake in the present study suggest that ADPN treatment promotes fatty acid oxidation in myoblasts.

Expectedly, ADPN treatment significantly increased the gene level of ATGL. According to research, a total of three enzymes are implicated in the complete hydrolysis of TG molecules in cellular lipid stores: ATGL selectively performs the first and rate-limiting step to hydrolyze TGs to generate diacylglycerols and nonestesterified fatty acids (NEFAs) [76,77]. The balance between TG hydrolysis and NEFA esterification tightly controls the cellular concentration of NEFAs [78–80]. ATGL deficiency in mice is associated with a severe reduction in liposolysis, leading to a significant increase in fat deposition in almost all tissues of the body, most notably in highly oxidized tissues such as testis, muscle, and the tubular system of the kidney [77,81]. Studies in human skeletal muscle have dissociated ATGL Ser406 phosphorylation from AMPK activation *in vitro* and *in vivo* during exercise [82]. All above results suggest that ADPN promotes fat uptake, lipolysis and subsequent oxidation of fatty acids, thereby reducing intracellular fat deposition.

### The AMPK signaling pathway is involved in the effects of adiponectin in chicken myoblasts

Skeletal muscle is an important site for glucose storage and lipid utilization. Lipids are stored as triacylglycerols in lipid droplets within skeletal muscle, called intermuscular triglycerides and are used as a substrate source for energy [64,83]. In mammals, AMPK is called a ‘metabolic sensor’ or ‘fuel gauge’ because it regulates not only ATP-consuming (anabolic; e.g., lipogenesis, cholesterol synthesis, protein synthesis) but also ATP-generating (catabolic; e.g., glycolysis, fatty acid oxidation) pathways, participating in the regulation of intermediary metabolism in response to intracellular energy demand [52,84]. In many cells, including cancer cells, AMPK is activated by adiponectin through interactions with ADPNR1/R2. AMPK activation via increased Thr^172^ phosphorylation leads to inactivation of ACC1/ACC2 and the resulting fall in malonyl-CoA relieves inhibition of CPT1, which increased import and β-oxidation of free fatty acids into mitochondria [48,85–87]. Therefore, we suspect that AMPK signaling pathway may also play a role in adiponectin regulation in chicken myoblasts. The protein expression of AMPK, ACC and CPT1 were detected using Western blot in myoblasts, and we found that ADPN with PA pretreatment activated the AMPK phosphorylation, suggesting the activation of AMPK pathway by ADPN. In spite of increased p-AMPK/T-AMPK by adiponectin in the presence of PA, there was no significant increase in p-ACC/T-ACC and CPT1 protein and activity. According to our unpublished data on shorter ADPN incubation times, the significant synchronous changes in p-AMPK/T-AMPK and p-ACC/T-ACC by ADPN in presence of PA were observed at 1 h, but not at 30 min and 6 h. Therefore, the reduced lipid content by ADPN observed at the time of 12 h in our present study was the cumulative effect of AMPK and its target genes over 12 h, and it is not easy to observe their synchronous changes. This may be due to the fact that ACC phosphorylation do not occur simultaneously with AMPK phosphorylation, which was demonstrated by Yoon et al. [88] that adiponectin increases fatty acid oxidation in skeletal muscle cells by sequential phosphorylation of AMPK, ACC and CPT1. To further verify the involvement of AMPK pathway, we treated myoblasts with Compound C, an AMPK inhibitor. The results showed that AMPK inhibition respectively alleviated and reversed the effects of adiponectin on ACC protein level and lipid content in chicken myoblasts. These results confirm that ADPN regulates lipid metabolism through the AMPK signaling pathway.

In summary, adiponectin reduced intracellular lipid content, likely by binding to ADPNR1/2, activating AMPK pathway, increasing cellular uptake of glucose and fatty acids, and promoting lipid oxidation. The regulating effect of adiponectin on chicken primary myoblasts is consistent with that of mammalian.

## Data Availability

All the data will be provided on reasonable request from the corresponding author.

## Abbreviations

CCK-8: cell counting kit-8
FAT: fatty acid translocase
GAPDH: glyceraldehyde 3-phosphate dehydrogenase
LCFA: long-chain fatty acid
NEFA: nonestesterified fatty acid

## References

[1] Brochu-Gaudreau K., Rehfeldt C., Blouin R., Bordignon V., Murphy B.D. and Palin M.F. (2010) Adiponectin action from head to toe. Endocrine 37, 11–32 10.1007/s12020-009-9278-8 20963555

[2] Maddineni S., Metzger S., Ocón O., Hendricks G. and Ramachandran R. (2005) Adiponectin gene is expressed in multiple tissues in the chicken: food deprivation influences adiponectin messenger ribonucleic acid expression. Endocrinology 146, 4250–4256 10.1210/en.2005-0254 15976057

[3] Cao Z., Li J., Luo L., Li X., Liu M., Gao M. et al. (2015) Molecular cloning and expression analysis of adiponectin and its receptors (AdipoR1 and AdipoR2) in the hypothalamus of the Huoyan goose during different stages of the egg-laying cycle. Reprod. Biol. *Endocrinol.* 13, 87 10.1186/s12958-015-0085-1 26251033 PMC4528393

[4] Goldstein B.J., Scalia R.G. and Ma X.L. (2009) Protective vascular and myocardial effects of adiponectin. Nat. Clin. Pract. Cardiovasc. Med. 6, 27–35 10.1038/ncpcardio1398 19029992 PMC2658652

[5] Kishore U. and Reid K.B. (2000) C1q: structure, function, and receptors. Immunopharmacology 49, 159–170 10.1016/S0162-3109(00)80301-X 10904115

[6] Maeda K., Okubo K., Shimomura I., Funahashi T., Matsuzawa Y. and Matsubara K. (1996) cDNA cloning and expression of a noveladipose specific collage n-like factor, apM1 (Adipose mostabundant gene transcript1). Biochem. Biophys. Res. Commun. 221, 286–289 10.1006/bbrc.1996.0587 8619847

[7] Hu E., Liang P. and SpiegIman B.M. (1996) AdipoQ is a noval adi-pose-specific gene dysregulated in obesity. J. Biol. Chem. 271, 10697–10703 10.1074/jbc.271.18.10697 8631877

[8] Jacobi S.K., Ajuwon K.M., Weber T.E., Kuske J.L., Dyer C.J. and Spurlock M.E. (2004) Cloning and expression of porcine adiponectin, and its relationship to adiposity, lipogenesis and the acute phase response. J. Endocrinol. 182, 133–144 10.1677/joe.0.1820133 15225138

[9] Yuan J., Liu W., Liu Z.L. and Li N. (2006) cDNA cloning, genomic structure, chromosomal mapping and expression analysis of ADIPOQ (adiponectin) in chicken. Cytogenet. Genome Res. 112, 148–151 10.1159/000087527 16276104

[10] Abhijit A.G., Amrita A.K. and Aniket A.K. (2018) Adiponectin: A potential therapeutic target for metabolic syndrome. Cytokine Growth Factor Rev. 39, 151–158 10.1016/j.cytogfr.2018.01.004 29395659

[11] Hendricks G.L., Hadley J.A., Krzysik-Walker S.M., Prabhu K.S., Vasilatos-Younken R. and Ramachandran R. (2009) Unique profile of chicken adiponectin, a predominantly heavy molecular weight multimer, and relationship to visceral adiposity. Endocrinology 150, 3092–3100 10.1210/en.2008-1558 19299452 PMC2703559

[12] Yan J., Tan X., Yan G.Y., Feng M., Yang H.L. and Sun C. (2014) Recombinant globular adiponectin inhibits lipid deposition by p38 MAPK/ATF-2 and TOR/p70 S6 kinase pathways in chicken adipocytes. Biochem. *Cell Biol.* 92, 53–60 24471918 10.1139/bcb-2013-0061

[13] Gan L., Yan J., Liu Z.J., Feng M. and Sun C. (2015) Adiponectin Prevents Reduction of Lipid-Induced Mitochondrial Biogenesis via AMPK/ACC2 Pathway in Chicken Adipocyte. J. Cell. Biochem. 116, 1090–1100 10.1002/jcb.25064 25536013

[14] Braun E.J. and Sweazea K.L. (2008) Glucose regulation in birds. Comp. Biochem. Physiol. Part B Biochem. Mol. Biol. 151, 1–9 10.1016/j.cbpb.2008.05.007 18571448

[15] Dupont J., Dagou C., Derouet M., Simon J. and Taouis M. (2004) Early steps of insulin receptor signaling in chicken and rat: apparent refractoriness in chicken muscle. Domest. Anim. Endocrinol. 26, 127–142 10.1016/j.domaniend.2003.09.004 14757185

[16] Halevy O., Geyra A., Barak M., Uni Z. and Sklan D. (2000) Early posthatch starvation decreases statellite cell proliferation and skeletal muscle growth in chicks. J. Nutr. 130, 858–864 10.1093/jn/130.4.858 10736342

[17] Fasshauer M., Klein J., Kralisch S., Klier M., Lössner U. and Blüher M. (2004) Growth hormone is a positive regulator of adiponectin receptor 2 in 3T3-L1 adipocytes. FEBS Lett. 558, 27–32 10.1016/S0014-5793(03)01525-4 14759511

[18] Neumeier M., Weigert J., Schäffler A., Wehrwein G., Müller-Ladner U., Schölmerich J. et al. (2006) Different effects of adiponectin isoforms in human monocytic cells. J. Leukoc. Biol. 79, 803–808 10.1189/jlb.0905521 16434692

[19] Yamauchi T., Kamon J., Ito Y., Tsuchida A., Yokomizo T., Kita S. et al. (2003) Cloning of adiponectin receptors that mediate antidiabetic metabolic effects. Nature 423, 762–769 10.1038/nature01705 12802337

[20] Maddineni S., Metzger S., Ocon O., Hendricks G. and Ramachandran R. (2005) Adiponectin gene is expressed in multiple tissues in the chickens: food deprivation influences adiponectin messenger ribonucleic acid expression. Endocrinology 146, 4250–4256 10.1210/en.2005-0254 15976057

[21] Ramachandran R., Ocon-Grove O.M. and Metzger S.L. (2007) Molecular cloning and tissue expression of chicken ADIPOR1 and ADIPOR2 complementary deoxyribonucleic acids. Domest. Anim. Endocrinol. 33, 19–31 10.1016/j.domaniend.2006.04.004 16697136

[22] Sintubin P., Decuypere E., Buyse J., Gertler A., Whitfield R. and Dridi S. (2011) Leptin and Cerulenin Differently Regulate Adiponectin Gene Expression in Chicken Liver and Hypothalamus. J. Microb. Biochem. Technol. 3, 67–72 10.4172/1948-5948.1000054

[23] Kadowaki T. and Yamauchi T. (2005) Adiponectin and adiponectin receptors. Endocr. Rev. 26, 439–451 10.1210/er.2005-0005 15897298

[24] Savage D.B., Petersen K.F. and Shulman G.I. (2005) Mechanisms of insulin resistance in humans and possible links with inflammation. Hypertension 45, 828–833 10.1161/01.HYP.0000163475.04421.e4 15824195

[25] Wolf G. (2003) Adiponectin, a regulator of energy homeostasis. Nutr. Rev. 61, 290–292 10.1301/nr.2003.aug.290-292 13677592

[26] Jeong K.J., Kim G.W. and Chung S.H. (2014) AMP-activated protein kinase: an emerging target for ginseng. J. Ginseng Res. 38, 83–88 10.1016/j.jgr.2013.11.014 24748831 PMC3986499

[27] Rasineni K. and Casey C.A. (2012) Molecular mechanism of alcoholic fatty liver. Indian J. Pharmacol. 44, 299–303 10.4103/0253-7613.96297 22701235 PMC3371448

[28] Xu A., Wang Y., Keshaw H., Xu L.Y., Lam K.S. and Cooper G.J. (2003) The fat-derived hormone adiponectin alleviates alcoholic and nonalcoholic fatty liver diseases in mice. J. Clin. Invest. 112, 91–100 10.1172/JCI200317797 12840063 PMC162288

[29] Satoh H., Nguyen M.T., Trujillo M., Imamura T., Usui I., Scherer P.E. et al. (2005) Adenovirus-mediated adiponectin expression augments skeletal muscle insulin sensitivity in male Wistar rats. Diabetes 54, 1304–1313 10.2337/diabetes.54.5.1304 15855314

[30] Ghadge A.A., Khaire A.A. and Kuvalekar A.A. (2018) Adiponectin: a potential therapeutic target for metabolic syndrome. Cytokine Growth Factor Rev. 39, 151–158 10.1016/j.cytogfr.2018.01.004 29395659

[31] Iwabu M., Yamauchi T., Okada-Iwabu M., Sato K., Nakagawa T., Funata M. et al. (2010) Adiponectin and AdipoR1 regulate PGC-1α and mitochondria by Ca^2+^ and AMPK/SIRT1. Nature 464, 1313–1319 10.1038/nature08991 20357764

[32] Cheng K.K., Lam K.S., Wang B. and Xu A. (2014) Signaling mechanisms underlying the insulin-sensitizing effects of adiponectin. Best Pract. Res. Clin. Endocrinol. Metab. 28, 3–13 10.1016/j.beem.2013.06.006 24417941

[33] Proszkowiec-Weglarz M., Richards M.P., Ramachandran R. and McMurtry J.P. (2006) Characterization of the AMP-activated protein kinase pathway in chickens. Comp. Biochem. Physiol. Part B Biochem. Mol. Biol. 143, 92–106 10.1016/j.cbpb.2005.10.009 16343965

[34] Yablonka-Reuveni Z. and Nameroff M. (1987) Skeletal muscle cell populations. Separation and partial characterization of fibroblast-like cells from embryonic tissue using density centrifugation. Histochemistry 87, 27–38 10.1007/BF00518721 3038797 PMC4096333

[35] Wang X.J., Song Z.G., Jiao H.C. and Lin H. (2012) Skeletal muscle fatty acids shift from oxidation to storage upon dexamethasone treatment in chickens. Gen. Comp. Endocrinol. 179, 319–330 10.1016/j.ygcen.2012.09.013 23036730

[36] Lillie R.D. and Fullmer H.M. (1977) Histopathologic technic and practical histochemistry (4th Edition). Biochem. Soc. Trans. 5, 558–559 10.1042/bst0050558a

[37] Zhang J.M., Sun Y.S., Zhao L.Q., Chen T.T., Fan M.N., Jiao H.C. et al. (2019) SCFAs-induced GLP-1 secretion links the regulation of gut microbiome on hepatic lipogenesis in chickens. Front. Microbiol. 10, 2176 10.3389/fmicb.2019.02176 31616396 PMC6775471

[38] Zhao L.Q., Liu S., Zhang Z.H., Zhang J.M., Jin X.Q., Zhang J. et al. (2020) Low and high concentrations of butyrate regulate fat accumulation in chicken adipocytes via different mechanisms. Adipocyte 9, 120–131 10.1080/21623945.2020.1738791 32163011 PMC7153540

[39] Liu L., Fu C.Y. and Li F.C. (2019) Acetate affects the process of lipid metabolism in rabbit liver, skeletal muscle and adipose tissue. Animals 9, 799 10.3390/ani9100799 PMC6826666 31615062

[40] Liu L., Zuo W.S. and Li F.C. (2019) Dietary addition of artemisia argyi reduces diarrhea and modulates the gut immune function without impacting growth performances of rabbits after weaning. J. Anim. Sci. 97, 1693–1700 10.1093/jas/skz047 30726960 PMC6447264

[41] Song M.Z., Lin X.Y., Zhao J.P., Wang X.J., Jiao H.C. and Lin H. (2020) High frequency vaccination-induced immune stress reduces bone strength with the involvement of activated osteoclastogenesis in layer pullets. Poult. Sci. 99, 734–743 10.1016/j.psj.2019.12.023 32029158 PMC7587667

[42] Wang H., Wang X.J., Zhao J.P., Jiao H.C. and Lin H. (2020) Low protein diet supplemented with crystalline amino acids suppressing appetite and apo-lipoprotein synthesis in laying hens. Anim. Feed Sci. Technol. 266, 114533 10.1016/j.anifeedsci.2020.114533

[43] Chen C., Wang H., Jiao H.C., Wang X.J., Zhao J.P. and Lin H. (2018) Feed habituation alleviates decreased feed intake after feed replacement in broilers. Poult. Sci. 97, 733–742 10.3382/ps/pex358 29253224

[44] Tang D.Z., Wu J.M., Jiao H.C., Wang X.J., Zhao J.P. and Lin H. (2019) The development of antioxidant system in the intestinal tract of broiler chickens. Poult. Sci. 98, 664–678 10.3382/ps/pey415 30289502

[45] Uerlings J., Song Z.G., Hu X.Y., Wang S.K., Lin H., Buyse J. et al. (2018) Heat exposure affects jejunal tight junction remodeling independently of adenosine monophosphate-activated protein kinase in 9-day-old broiler chicks. Poult. Sci. 97, 3681–3690 10.3382/ps/pey229 29901744

[46] Liu L., Xu S.H., Wang X.J., Jiao H.C. and Lin H. (2016) Peripheral insulin doesn't alter appetite of broiler chicks. Asian-Australas. J. Anim. Sci. 29, 1294–1299 10.5713/ajas.15.0674 26954230 PMC5003990

[47] Wang R.M., Zhao J.P., Wang X.J., Jiao H.C., Wu J.M. and Lin H. (2018) Fibroblast growth factor 23 mRNA expression profile in chickens and its response to dietary phosphorus. Poult. Sci. 97, 2258–2266 10.3382/ps/pey092 29688456

[48] Yamauchi T., Kamon J., Waki H., Terauchi Y., Kubota N., Hara K. et al. (2001) The fat-derived hormone adiponectin reverses insulin resistance associated with both lipoatrophy and obesity. Nat. Med. 7, 941–946 10.1038/90984 11479627

[49] Maeda K., Ishihara K., Miyake K., Kaji Y., Kawamitsu H., Fujii M. et al. (2005) Inverse correlation between serum adiponectin concentration and hepatic lipid content in Japanese with type 2 diabetes. Metab.-Clin. Exp. 54, 775–780 10.1016/j.metabol.2005.01.020 15931613

[50] Gan L., Yan J., Liu Z.J., Fen M. and Sun C. (2015) Adiponectin prevents reduction of lipid-induced mitochondrial biogenesis via AMPK/ACC2 pathway in chicken adipocyte. J. Cell. Biochem. 116, 1090–10100 10.1002/jcb.25064 25536013

[51] Yamauchi T., Nio Y., Maki T., Kobayashi M., Takazawa T., Iwabu M. et al. (2007) Targeted disruption of AdipoR1 and AdipoR2 causes abrogation of adiponectin binding and metabolic actions. Nat. Med. 13, 332–339 10.1038/nm1557 17268472

[52] Iwabu M., Okada-Iwabu M., Yamauchi T. and Kadowaki T. (2015) Adiponectin/adiponectin receptor in disease and aging. Npj Aging Mech. Dis. 1, 15013 10.1038/npjamd.2015.13 28721260 PMC5514982

[53] Jung U.J. and Choi M.S. (2014) Obesity and its metabolic complications: the role of adipo-kines and the relationship between obesity, inflammation, insulin resistance, dyslipidemia and nonalcoholic fatty liver disease. Int. J. Mol. Sci. 15, 6184–6223 10.3390/ijms15046184 24733068 PMC4013623

[54] Ceddia R.B., Somwar R., Maida A., Fang X., Bikopoulos G. and Sweeney G. (2005) Globular adiponectin increases GLUT4 translocation and glucose uptake but reduces glycogen synthesis in rat skeletal muscle cells. Diabetologia 48, 132–139 10.1007/s00125-004-1609-y 15619075

[55] Schaffer J.E. and Lodish H.F. (1994) Expression cloning and characterization of a novel adipocyte long chain fatty acid transport protein. Cell 79, 427–436 10.1016/0092-8674(94)90252-6 7954810

[56] Wu Q., Ortegon A.M., Tsang B., Doege H., Feingold K.R. and Stahl A. (2006) FATP1 is an insulin-sensitive fatty acid transporter involved in diet-induced obesity. Mol. Cell. Biol. 26, 3455–3467 10.1128/MCB.26.9.3455-3467.2006 16611988 PMC1447434

[57] Wu Q., Kazantzis M., Doege H., Ortegon A.M., Tsang B., Falcon A. et al. (2006) Fatty acid transport protein 1 is required for nonshivering thermogenesis in brown adipose tissue. Diabetes 55, 3229–3237 10.2337/db06-0749 17130465

[58] Kazantzis M. and Stahl A. (2012) Fatty acid transport proteins, implications in physiology and disease. Biochim. Biophys. Acta 1821, 852–857 10.1016/j.bbalip.2011.09.010 21979150 PMC3274620

[59] Stahl A., Gimeno R.E., Tartaglia L.A. and Lodish H.F. (2001) Fatty acid transport proteins: a current view of a growing family. Trends Endocrinol. Metab. 12, 266–273 10.1016/S1043-2760(01)00427-1 11445444

[60] Semenkovich C.F. (1997) Regulation of fatty acid synthase (FAS). Prog. Lipid Res. 36, 43–53 10.1016/S0163-7827(97)00003-9 9373620

[61] Smith S. (1994) The animal fatty acid synthase: one gene, one polypeptide, seven enzymes. FASEB J. 8, 1248–1259 10.1096/fasebj.8.15.8001737 8001737

[62] De Silva G.S., Desai K., Darwech M., Naim U., Jin X., Adak S. et al. (2019) Circulating serum fatty acid synthase is elevated in patients with diabetes and carotid artery stenosis and is LDL-associated. Atherosclerosis 287, 38–45 10.1016/j.atherosclerosis.2019.05.016 31202106 PMC6707847

[63] Hudgins L.C., Hellerstein M.K., Seidman C.E., Neese R.A., Tremaroli J.D. and Hirsch J. (2000) Relationship between carbohydrate-induced hypertriglyceridemia and fatty acid synthesis in lean and obese subjects. J. Lipid Res. 41, 595–604 10.1016/S0022-2275(20)32407-X 10744780

[64] Kiens B. (2006) Skeletal muscle lipid metabolism in exercise and insulin resistance. Physiol. Rev. 86, 205–243 10.1152/physrev.00023.2004 16371598

[65] Wahli W., Braissant O. and Desvergne B. (1995) Peroxisome proliferator activated receptors: transcriptional regulators of adipogenesis, lipid metabolism and more. Chem. Biol. 2, 261–266 10.1016/1074-5521(95)90045-4 9383428

[66] Berger J., Tanen M., Elbrecht A., Hermanowski-Vosatka A., Moller D.E., Wright S.D. et al. (2001) Peroxisome proliferator-activated receptor γ ligands inhibit adipocyte 11beta-hydroxysteroid dehydrogenase type 1 expression and activity. J. Biol. Chem. 276, 12629–12635 10.1074/jbc.M003592200 11278270

[67] Rangwala S.M., Rhoades B., Shapiro J.S., Rich A.S., Kim J.K., Shulman G.I. et al. (2003) Genetic modulation of PPARγ phosphorylation regulates insulin sensitivity. Dev. Cell 5, 657–663 10.1016/S1534-5807(03)00274-0 14536066

[68] Kliewer S.A., Forman B.M., Blumberg B., Ong E.S., Borgmeyer U., Mangelsdorf D.J. et al. (1994) Differential expression and activation of a family of murine peroxisome proliferator-activated receptors. Proc. Natl. Acad. Sci. U.S.A. 91, 7355–7359 10.1073/pnas.91.15.7355 8041794 PMC44398

[69] Kahn B.B. and Flier J.S. (2000) Obesity and insulin resistance. J. Clin. Invest. 106, 473–481 10.1172/JCI10842 10953022 PMC380258

[70] Lemberger T., Desvergne B. and Wahli W. (1996) Peroxisome proliferator-activated receptors: a nuclear receptor signaling pathway in lipid physiology. Annu. Rev. Cell Dev. Biol. 12, 335–363 10.1146/annurev.cellbio.12.1.335 8970730

[71] Auboeuf D., Rieusset J., Fajas L., Vallier P., Frering V., Riou J.P. et al. (1997) Tissue distribution and quantification of the expression of mRNAs of peroxisome proliferator-activated receptors and liver X receptor in humans. Diabetes 46, 1319–1327 10.2337/diab.46.8.1319 9231657

[72] Braissant O., Foufelle F., Scotto C., Dauça M. and Wahli W. (1996) Differential expression of peroxisome proliferator-activated receptor (PPARs): tissue distribution of PPAR-alpha, -beta, and -gamma in the adult rat. Endocrinology 137, 354–366 10.1210/endo.137.1.8536636 8536636

[73] Leone T.C., Weinheimer C.J. and Kelly D.P. (1999) A critical role for the peroxisome proliferator-activated receptor α (PPAR-α) in the cellular fasting response: the PPARα-null mouse as a model of fatty acid oxidation disorders. Proc. Natl. Acad. Sci. U.S.A. 96, 7473–7478 10.1073/pnas.96.13.7473 10377439 PMC22110

[74] Motojima K., Passilly P., Peters J.M., Gonzalez F.J. and Latruffe N. (1998) Expression of putative fatty acid transporter genes are regulated by peroxisome proliferator-activated receptor α and γ activators in a tissue- and inducer-specific manner. J. Biol. Chem. 273, 16710–16714 10.1074/jbc.273.27.16710 9642225

[75] Vosper H., Khoudoli G.A., Graham T.L. and Palmer C.N. (2002) Peroxi-some proliferator-activated receptor agonists, hyperlipidaemia, and atherosclerosis. Pharmacol. Ther. 95, 47–62 10.1016/S0163-7258(02)00232-2 12163127

[76] Zimmermann R., Strauss J.G., Haemmerle G., Schoiswohl G., Birner-Gruenberger R., Riederer M. et al. (2004) Fat mobilization in adipose tissue is promoted by adipose triglyceride lipase. Science 306, 1383–1386 10.1126/science.1100747 15550674

[77] Lass A., Zimmermann R., Oberer M. and Zechner R. (2011) Lipolysis - a highly regulated multi-enzyme complex mediates the catabolism of cellular fat stores. Prog. Lipid Res. 50, 14–27 10.1016/j.plipres.2010.10.004 21087632 PMC3031774

[78] Duncan R.E., Ahmadian M., Jaworski K., Sarkadi-Nagy E. and Sul H.S. (2007) Regulation of lipolysis in adipocytes. Annu. Rev. Nutr. 27, 79–101 10.1146/annurev.nutr.27.061406.093734 17313320 PMC2885771

[79] Langin D. (2006) Control of fatty acid and glycerol release in adipose tissue lipolysis. C. R. Biol. 329, 598–607, (discussion 653-655) 10.1016/j.crvi.2005.10.008 16860278

[80] Zechner R., Strauss J.G., Haemmerle G., Lass A. and Zimmermann R. (2005) Lipolysis: pathway under construction. Curr. Opin. Lipidol. 16, 333–340 10.1097/01.mol.0000169354.20395.1c 15891395

[81] Haemmerle G., Lass A., Zimmermann R., Gorkiewicz G., Meyer C., Rozman J. et al. (2006) Defective lipolysis and altered energy metabolism in mice lacking adipose triglyceride lipase. Science 312, 734–737 10.1126/science.1123965 16675698

[82] Mason R.R., Meex R.C.R., Lee-Young R., Canny B.J. and Watt M.J. (2012) Phosphorylation of adipose triglyceride lipase Ser404 is not related to 5'-AMPK activation during moderate-intensity exercise in humans. Am. J. Physiol.-Endocrinol. Metab. 303, 534–541 10.1152/ajpendo.00082.2012 22713505

[83] van Loon L.J. (2004) Use of intramuscular triacylglycerol as a substrate source during exercise in humans. J. Appl. Physiol. 97, 1170–1187 10.1152/japplphysiol.00368.2004 15358749

[84] Kahn B.B., Alquier T., Carling D. and Hardie D.G. (2005) AMP-activated protein kinase: ancient energy gauge provides clues to modern understanding of metabolism. Cell. Metab. 1, 15–25 10.1016/j.cmet.2004.12.003 16054041

[85] Deepa S.S. and Dong L.Q. (2009) APPL1: role in adiponectin signaling and beyond. Am. J. Physiol. Endocrinol. Metab. 296, E22–E36 10.1152/ajpendo.90731.2008 18854421 PMC2636986

[86] Karbowska J. and Kochan Z. (2006) Role of adiponectin in the regulation of carbohydrate and lipid metabolism. J. Physiol. Pharmacol. 57, 103–113 17228091

[87] Mao X., Kikani C.K., Riojas R.A., Langlais P., Wang L., Ramos F.J. et al. (2006) APPL1 binds to adiponectin receptors and mediates adiponectin signalling and function. Nat. Cell Biol. 8, 516–523 10.1038/ncb1404 16622416

[88] Yoon M.J., Lee G.Y., Chung J.J., Ahn Y.H., Hong S.H. and Kim J.B. (2006) Adiponectin increases fatty acid oxidation in skeletal muscle cells by sequential activation of AMP-activated protein kinase, p38 mitogen-activated protein kinase, and peroxisome proliferator-activated receptor alpha. Diabetes 55, 2562–2570 10.2337/db05-1322 16936205

